# “I want control and I imagine I have it” experiences of control and loss of control among people with gambling problems in Sweden

**DOI:** 10.1080/17482631.2025.2530019

**Published:** 2025-07-08

**Authors:** Ellen Gerle, Tove Lundberg, Björn Hofvander, Anders Håkansson

**Affiliations:** a Faculty of Medicine, Department of Clinical Sciences Lund, Psychiatry, Lund University, Lund, Sweden; b Department of Psychology, Lund University, Lund, Sweden; c Lund Clinical Research on Externalizing and Developmental Psychopathology, Department of Clinical Sciences Lund, Lund University, Lund, Sweden

**Keywords:** Gambling, self-control, interpretative phenomenological analysis, qualitative, control

## Abstract

**Introduction:**

Gambling harm is a widespread phenomenon affecting an increasing number of individuals. Understanding the concept of self-control is central to comprehending the nature of gambling problems.

**Methods:**

Semi-structured in-depth interviews were conducted with ten individuals recruited through peer support associations in Sweden. The data was analysed using Interpretative Phenomenological Analysis.

**Results:**

Three group themes were identified: (Un)predictability and (Un)safety: The Meanings of Being in or Out of Control, Inside “the Bubble”: Navigating (No) Control in and Through Gambling, and A Delicate Act: “Regaining” Control of an Uncontrolled Life. Gambling was described as part of a negative spiral in which individuals in challenging life situations attempt to create and recreate experiences of control through gambling. The more negative consequences gambling had on everyday life and relationships, the greater the need to retreat into a protective “bubble.”

**Discussion:**

Being in and out of control are universal human experiences through which we can better understand the maintenance of severe gambling problems. Between these two experiences, there is also a false sense of control that does not correspond to experiences previously described in the literature.

## Introduction

Self-control refers to the capacity to regulate one’s behaviour in accordance with personal ideals, values, morals, and societal expectations, thereby supporting the pursuit of long-term goals (Baumeister et al., 2007). As such, it plays a vital role in enabling individuals to function effectively within society (DeLisi, 2014). Impaired control is an essential concept of addictive behaviours generally (Corless & Dickerson, 1989) and has, related to gambling, been understood as “difficulties in resisting impulses to gamble” (Corless & Dickerson, 1989, p. 1527).

Loss of control is a crucial aspect in understanding gambling behaviour, and further research on this phenomenon is needed (Gong & Zhu, 2019). Bergen et al. (2012) argue that many studies on control lack clear conceptualizations of self-control, highlighting the need for more nuanced discussions about how loss of control should be understood within gambling research. Moreover, it is considered one of the defining features of gambling disorders (American Psychiatric Association & American Psychiatric Association, 2013).

In this study, we employed a hermeneutic phenomenological and idiographic approach to examine the nuances of self-control and loss thereof. We conducted in-depth interviews with participants recruited from Swedish peer support groups to understand how these individuals perceive and experience self-control and loss thereof. Before presenting our results, we briefly introduce the Swedish gambling context and review previous approaches to self-control in relation to gambling.

The point prevalence of gambling problems has been estimated to be between 0.1–5.8%, and lifetime prevalence has been estimated to be between 0.7% and 6.5% (Calado & Griffiths, 2016). Problems with gambling are shown to contribute to negative social, health, and financial consequences and are often experienced together with other mental health challenges (Hodgins et al., 2011). Only a minority of people who experience gambling problems seek help (Braun et al., 2014) and among those who do, impaired control is a central part of their experience of gambling (Corless & Dickerson, 1989). The need to regain control is an important part of the help-seeking process (De Vos et al., 2021) and one commonly reported barrier to seeking help is the shame and fear of stigma (Hing et al., 2014; Pchajek et al., 2022; Suurvali et al., 2009).

In the realm of problematic gambling, self-control can be defined as a person’s capacity to alter or suppress their impulse to gamble (Thurm et al., 2023). Blaszczynski and Nower (2002) define impaired control as “repeated unsuccessful attempts to resist the urge in the context of a genuine desire to cease, as the central, diagnostic and foundational feature of pathological gambling” (p. 488). Dickerson and O’Connor (2006) discuss impaired control in terms of the deterioration of individuals’ ability to manage their time and money spent on gambling. This impairment is characterized by behaviours such as ‘gambling for longer than intended”, “spending more than planned”, and “spending beyond one’s means” (p. 26).

Nolan et al. (2024) explore the concept of self-control through three primary approaches. The first approach involves setting personal boundaries, such as limiting the frequency, duration, and financial expenditure on gambling activities. The second approach emphasizes mindful gambling, where individuals consciously consider the risks and make informed choices. The third approach acknowledges the challenges in maintaining self-control, particularly for those with higher scores on the Problem Gambling Severity Index, despite employing various strategies.

Concrete strategies for self-control that have been examined in relation to gambling include setting a predetermined spending limit in advance, maintaining a record of expenditures, and limiting alcohol consumption (Currie et al., 2020). To restrict the amount of time spent playing, consider the negative consequences of excessive gambling, and limit the frequency of gambling activities have also been examined. Further strategies involve restricting access to additional cash, engaging in gambling activities with friends and/or family present, and limiting cannabis consumption (Currie et al., 2020)

Participants in a study by Flores-Pajot et al. (2021) identified various factors that impacted adherence to self-control strategies, which either augmented or diminished their predetermined gambling limits, or affected both. For example, social influences and the outcomes of winning or losing money either motivated participants to refrain from gambling or prompted them to continue. Additionally, misconceptions about gambling, such as the belief in being lucky, also played a role in the utilization of self-control strategies.

Several approaches emphasize personal responsibility, rational decision-making, and impulse regulation, framing control as an internal, stable trait or skill. The aim of this study is to fully grasp self-control in terms of individuals’ experiences. To do this the experiences need to be described and contextualized in relation to everyday life and the individuals’ relationship with oneself and others.

Generally, it has been argued that self-control contributes to several positive outcomes in life, and has been understood as “the ability to override or change one’s inner responses, as well as to interrupt undesired behavioural tendencies and refrain from acting on them” (Tangney et al., 2004, p. 275). Self-control has been investigated in numerous disciplines, such as psychology, philosophy, and neuroscience (Levy, 2013; Lopez-Gonzalez et al., 2018). Gong and Zhu (2019) highlight that the relationship between gambling and self-control has rarely been examined in existing research. We emphasize that self-control in relation to gambling is an emerging topic that warrants further investigation.

One exploration of control has focused on gamblers’ illusion of control over outcomes in which they lack control (Langer, 1975; Presson & Benassi, 1996). Gamblers typically overestimate their control and create causal relationships between their behaviour and uncontrollable events (Lopez-Gonzalez et al., 2018).

Based on the argument that many studies of control suffer from the lack of clear conceptualizations, Bergen et al. (2012) suggest that we interpret self-control according to the strength model. In the strength model self-control is understood to be similar to a muscular strength and is interpreted as a limited willpower where exercising self-control in one domain leaves less self-control to other domains. While advances in neuropsychology as well as developments in personality research on self-control are essential to our understanding of failure of self-control, Bergen et al. (2012) argue that the conceptualization of self-control as a strength can provide a framework for explaining how self-control functions in the individual and how it can be improved. The strengths model is dominant but has also been criticized, for example it has been shown that demanding tasks do not reduce the ability to perform self-control (Thurm et al., 2023). Although the strengths model offers a clear and concrete approach to how we can understand self-control, it lacks a broader and more developed in-depth insight into how the individual relates to the experience and perception of self-control and failures in self-control. To fully understand control in relation to gambling control needs to be understood in the context and relationship to life in general.

Although this offers insight into self-control, there is a need for more in-depth insight into how individuals relate to the experience and perception of self-control and failures in self-control. Reith and Dobbie (2012) described a kind of dual self in which one part of the person wants to continue gambling, while the other part tries to abstain. However, what is still not described is the motives and mechanisms behind a person’s gambling. Is there a motive for repeatedly losing control, even when the negative consequences are devastating? Blaszczynski and Nower (2002) presented a pathway model with three different paths to gambling problems: conditioning and behavioural factors, emotional vulnerability, and impulsiveness and anti-sociality. The’ pathways’ model does not characterize these pathways as being mutually exclusive but rather it views the risk factors as cumulative. Turner et al. (2006) indicate that most pathological gamblers are on more than one path. Although the pathway model offers a substantial explanation of the different paths to gambling problems, it has little to say about the perceptions of the individual who experiences and wanders on those paths. An in-depth qualitative study could contribute to a more detailed understanding of how control is experienced in relation to gambling.

van Schalkwyk et al. (2022) reason that the core concept of responsible gambling posits gambling products, services, and practices as inherently harmless, with harm arising from misuse or abnormal gambling. According to this perspective, the risk of harm is attributed to individuals not gambling responsibly, which is seen as a result of their lack of control, irresponsibility, or poor decision-making (van Schalkwyk et al. (2022). Thus, the right way to gamble is controlled as for the responsible gambler, and the wrong way, as for the problem gambler, is uncontrolled. van Schalkwyk et al. (2022) argue that “what constitutes responsible or safe gambling often remains ambiguously defined” (p 2). Hodgins et al. (2021) contends that the responsible gambling narrative oversimplifies the causes of harm related to gambling, failing to accurately capture the various factors that influence individuals’ decision-making processes.

In clinical practice, the concept of control is often central to how gambling problems are assessed and treated. However, framing control merely as something one either possesses or lacks risks oversimplifying the lived complexity of individuals’ experiences. This study is grounded in the need to develop a more nuanced understanding of how control is felt, negotiated, and narrated in everyday life. By exploring the subtleties of how individuals describe their struggles with control, often marked by ambivalence, shame, and efforts at self-regulation, we aim to contribute to a language that resonates with the realities of those affected. Such insights are crucial for informing interventions that are not only effective but also attuned to the specific and holistic contexts in which people live. As such, this work is part of a broader intellectual effort to bridge experiential knowledge with clinical applicability, and to move beyond abstract dichotomies towards a more situated understanding of what it means to live with and attempt to regain control.

In recent decades, several measures have been proposed to counteract the negative effects of gambling harms, a discussion that is also evident in Sweden (Håkansson et al., 2022). In 2018, gambling disorder was included in the Swedish legislation regulating social services, and today, local municipal social services and regional health care providers share the responsibility for the treatment of people with addictions, including gambling disorder (Håkansson et al., 2022). In 2019, the Swedish Gambling Act (Spellag 2018:1138) came into force, introducing licencing for domestic gambling vendors in Sweden. On one hand, this legislation meant that the previous gambling monopoly which had been valid for many gambling types—along with a low number of other gambling operators in specific gambling types—was abolished. Thus, while this meant that the establishment of private gambling operators was strongly liberalized, on the other hand, the previous monopoly or oligopoly situation in reality had been characterized for several years by a large market share from overseas online gambling operators operating onto the Swedish market without being formally legal here. These represented, for example online casino, by far the most common gambling type in treatment seekers (Håkansson et al., 2017), among individuals with gambling problems (Håkansson & Widinghoff, 2020), and in television commercials (Håkansson & Widinghoff, 2019), even before this gambling type was even formally legal in this setting.

Also, this novel licence market system also introduced new regulations which legally excluded international gambling operators without a Swedish licence from operating on the Swedish market, something that had been growing considerably during the years before. In total, this change meant that the number of legal operators, most commonly in online casino and sports betting, increased considerably on the Swedish market (Andersson et al., 2022). In addition, and with relevance to the topic of self-control in gambling, the new gambling act in Sweden also involved the introduction in 2019 of a multi-operator, nationwide self-exclusion system (“Spelpaus”) for all licenced gambling, both for online and land-based gambling on these licenced operators (Håkansson & Widinghoff, 2020). Thus, this is a system where an individual can—without using separate self-exclusion tools of each gambling operator—self-exclude from all operators involved in online casino gambling, sports or horse race betting, online or land-based bingo, land-based electronic gambling machine gambling, and online casino gambling, for a period of either one, three, six, or twelve months. The only gambling types not included in the self-exclusion service are lottery tickets bought in kiosques or grocery stores, and the low-stake table casino games available in some restaurants and night clubs but which are limited in access and the level of money that can be inserted, and which are rare in the reporting of gambling problems in Sweden (Miles et al., 2023). One part of the rationale behind self-exclusion is to help the person deal with a loss of control over their gambling.

### The current study

As control is a core concept in gambling problems, it follows that exploring experiences of control may be crucial in continuing to develop an understanding of the underlying processes not only of gambling problems, but also of addiction in general (Corless & Dickerson, 1989). The primary aim of Interpretative Phenomenological Analysis (IPA) is to explore human experiences and provide deeper insights into the complex experiences of those affected (Smith, 2004). The present study did not include participants with any pre-determined definition of control. Instead, they were encouraged to formulate their own understanding of being in and out of control.

This study focuses on participants’ experiences of control both in gambling as well as in everyday life and self-image. This allowed us to highlight the relationships between these areas of control and their implications for each other. Through in-depth interviews, this study aims to provide a deeper understanding of the complexity of the experiences of gambling and its relationship with oneself and others. To understand the role of control in gambling problems, we must consider it through the horizons of meaning that shape an individual’s experience. Gambling problems are not isolated phenomena; they are deeply embedded in an individual’s life world. By examining participants’ experiences and interpreting the subtle nuances of their perceptions, we can reach a deeper understanding of the complex dynamics of control. By acknowledging and exploring these horizons of meaning, we can gain richer insight into the forces driving gambling problems and the possibilities for regaining control over one’s life.

The research question for this study was as follows: How is control experienced by individuals with a history of gambling problems?

## Method

### Study methodology

This study used IPA (Smith, 2004) as a framework for study design, data collection, and analysis. IPA was chosen to enable an in-depth exploration of participants’ thoughts, feelings, and interpretations of their experiences of control. IPA aims to explore individuals’ personal lived experiences and how they make sense of them (Smith, 2004). IPA is phenomenological in its focus on individuals’ perceptions of objects and events and hermeneutic in its concern with the interpretative process where the analyst is trying to understand the individual who is trying to understand (Smith, 2004). IPA is also idiographic in the sense that it focuses on one case in a detailed examination until “some degree of closure or gestalt” has been accomplished to then move on to the next case (Smith, 2004, p. 41). Small samples were recommended, between 5–10 participants were suggested by Smith (2004).

### Ethics

Study approved by the regional ethical review board in Lund (No 2019–01454) and was conducted in accordance with the ethical principles of the Declaration of Helsinki (World Medical Association, 2013). Before the interviews were booked, participants received written information about the study, describing confidentiality and their right to discontinue participation. Any data that would risk identifying a participant was anonymized before the researchers other than the interviewer accessed the data. De-identification was given special consideration as the participants were recruited from the same associations. Participants were given a number to report the data, see Table I.

**Table I.** Participants and characteristics.

Participant number	Score on NODS	Living situation	Children
1	10	Cohabiting with partner	Living with children
2	10	Cohabiting with partner	Child moved out
3	10	Cohabiting with partner	Living with children
4	8	Cohabiting with partner	Living with child
5	10	Living single	Children moved out
6	8	Living single	Children moved out
7	8	Cohabiting with partner	Living with children
8	10	Cohabiting with partner	Living with children
9	10	Living single	Living with children
10	10	Living single	Living with children

*Note*. Participant numbers were assigned to protect confidentiality.

### Recruitment

Participants were recruited through the National Association of Gambling Addicts (in Swedish Spelberoendes riksförbund, SBRF) and Gamblers Anonymous (GA), two national mutual support societies. The first author visited the staff at the SBRF and then took part in a member meeting. Contact with a representative from GA was obtained through telephone calls. Societies shared a short, informative text on the study in their social media platforms and meetings. People with personal experiences of gambling problems were invited to an interview focusing on gambling and perceptions of control. Of the 13 people who expressed their interests, 10 took part in the interviews. The remaining three individuals cited personal circumstances as the reason for not participating.

The participants completed the National Opinion Research Center DSM-IV Screen for Gambling Problems (NODS) before the interviews began. In the NODS, total scores can range from 0 to 10, with higher scores indicating more serious gambling problems (Wickwire et al., 2008). Scores between 1 and 2 indicate risk, 3–4 indicate problem gambling, and scores over 5 suggest pathological gambling (Wickwire et al., 2008). The scores varied between 8 and 10 (Mdn_score_ = 10), suggesting that the participants’ experiences were clinically relevant, the scores are displayed in Table I.

#### Participants

The participants were aged between 35 and 67 years (Mdn_age_ = 46). Two participants were women, and eight were men. They live in different parts of Sweden. Most were cohabiting with their partners. All participants had children, and some of them had children still lived at home. Several of them had children with different types of difficulties who needed to seek help. Most of them were currently employed. The participants recovered from severe gambling problems and reached different stages of their journey with multiple relapses.

#### Procedure

The first author conducted semi-structured interviews based on the interview protocol. The interview questions concerned living situations, gambling history, and experiences of control in relation to gambling in everyday life. The interview design used a continuum to depict control, with one end showing a lot of control and the other showing little control, or that control was outside oneself. An example from the interview guide is: “The focus of this study is on different types of control related to gambling. Control can be described as a line where one end indicates full control and the other end indicates no control. (*Here we use an image of a continuum between ‘in control’ and ‘not in control/control is beyond me*’.) To get an idea of what it looks like for you, if we go to this end (*pointing to full control*), can you describe an everyday situation where you experience it, it does not have to be gambling-related?” Can you provide more examples of areas or situations in your life where you feel you are in control? *Ask for Concrete examples”*.

Before the interview, the participants signed an informed consent form, and NODS was administered. The interviews were conducted digitally using a programme in which the interviewer and the participant communicated both video and sound. The interviews lasted between 51 and 95 minutes (Mdn_time_ = 74 minutes). Interviews were recorded using a separate audio device and transcribed verbatim.

To enhance the linguistic clarity of the manuscript, Microsoft Copilot (OpenAI), an AI-based language model, was used for language editing. The final text was carefully reviewed and revised by the authors to ensure accuracy and integrity.

#### Analysis

Data analysis was guided by the principles of IPA, as recommended by Smith et al. (2009, 2022). Data immersion began with the first author listening to the interviews, and then reading and re-reading the transcripts. The research question, guided by the IPA, focused on how participants made sense of their experiences. While reading and re-reading the data, the first author commented on each individual transcript. The next step was to formulate experiential statements (Smith et al., 2022). This analytic move was conducted by shifting the focus from the transcript to exploratory notes from the previous step. This move is one way of working with the hermeneutic circle, moving between the whole and the parts of the whole. The statements are built on the meaning of participants’ words, as well as on analysts’ interpretation. The next step was to map out how experiential statements fit together into Personal Experiential Themes, themes derived from analysing the experiences of an individual reflecting the distinct ways in which the individual interprets and understands their experiences. One example from the PETs was: “I was caught off-guard by a question I should have known the answer to. But I wasn’t prepared for that question to come up. And I noticed, it was very hard on me. I didn’t like that kind of curveball at all.” In the next step, the first author moved on to the next case by repeating this process. After this step, the work of looking for patterns across cases followed, and Group Experiential Themes were formulated, highlighting areas where different individuals’ experiences converge or diverge, providing a broader understanding. The analytic process involved the identification of a range of preliminary themes, some of which were ultimately excluded from the final thematic structure. These themes, while initially appearing salient, were either insufficiently grounded in the data or lacked the depth and recurrence necessary for inclusion. One such theme was: *Control in Relation to Professional Life*: Several participants made passing references to how their experiences of control, or lack thereof, extended into their professional lives. However, these accounts were often brief and lacked the experiential richness required for deeper interpretative engagement. As such, this theme was not retained in the final analysis. Another discarded theme was *Gambling—The Invisible Addiction*: This theme captured participants’ reflections on the hidden and socially unacknowledged nature of their gambling problems. Although conceptually compelling, it overlapped with themes related to secrecy, stigma, and identity management, and was therefore subsumed within broader interpretative categories. While these themes were not developed further, they played a valuable role in shaping the analytic process and refining the focus of the final thematic structure.

The process was guided by the guidelines suggested by Elliott et al. (1999), for example, following the “grounding in examples” where we aimed to provide rich examples from the data in a way that offered the reader the opportunity to conceptualize possible alternative meanings. We have endeavoured to capture the nuanced and textured nature of participants’ lived experiences of control in the context of gambling problems. Rather than presenting control as a fixed or binary state, we sought to illuminate how it was experienced as shifting, fragile, and often illusory—embedded in everyday practices, emotions, and self-understandings. In doing so, we aimed to articulate aspects of control that are often difficult to express, yet deeply relevant for understanding the psychological and relational dynamics that may inform both the development of, and recovery from, gambling problems.

Through the process of analysis, the first author wrote down thoughts and ideas, keeping track of the analytic work. In accordance with the IPA approach outlined by Smith et al. (2022), the research team engaged in continuous discussions throughout the interview process and the subsequent analysis. These discussions focused on the participants’ experiences, relevant scientific literature, and clinical insights into gambling harms and perceptions of control. In this process, the experiences of the research team, including expertise in addiction and gambling problems, clinical psychology, and qualitative methodology, were important in addressing different nuances in the data. Additionally, various interpretations of control, as well as methodological and psychological aspects, were explored, in line with Elliott et al. (1999) recommendations for credibility checks.

In accordance with a phenomenological perspective, the pre-understandings of the research team, comprising three psychologists and one psychiatrist, were continuously discussed (Smith et al., 2022). The research team includes individuals with both insider and outsider perspectives within the field, which has significantly shaped our reflexive approach to the study. One team member is a physiatrist specializing in addiction medicine with specific clinical experience in gambling disorder, offering an insider’s view grounded in direct patient care and systemic knowledge of addiction services. Another is a psychologist with a background in neuropsychology and forensic psychiatry, contributing expertise in cognitive functioning and legal-psychiatric contexts. A third member brings experience from working with traumatized children, offering a developmental and trauma-informed lens. The fourth member is a senior lecturer in qualitative methodologies and has specialized knowledge in minority stress and contributes a norm-critical and intersectional perspective on identity and marginalization. This diversity of professional backgrounds—some embedded within the field and others approaching from adjacent domains—has enabled a multifaceted interpretation of the data. Throughout the research process, we engaged in continuous reflexive dialogue, particularly during group discussions, to critically examine how our respective positions and preconceptions may have influenced the analysis. In line with Elliott et al. (1999) criteria for quality in qualitative research, these discussions served as a form of *credibility check*, allowing us to challenge and refine our interpretations collaboratively, thereby enhancing the trustworthiness of our findings.

The first author also maintained reflexive notes throughout the data collection, group discussions, and analysis processes, documenting thoughts and emotional responses related to the research journey. One concrete outcome of this reflexive practice was a growing awareness of how participants’ narratives often carried elements of shame and confession, particularly when describing their struggles with gambling. Drawing on clinical experience with trauma-focused therapy, the first author reflected on how these narratives resembled therapeutic processes of disclosure and emotional regulation. This insight influenced the analytical lens by highlighting how participants’ accounts were not only descriptive but also performative positioning themselves in relation to moral and social expectations. As a result, the final analysis paid particular attention to how shame, accountability, and the need for recognition shaped the way participants constructed their stories, especially in relation to efforts to regain control.

## Results

The three Group Experiential Themes (GETs) were connected to every interview except for GET 3 *A delicate act: “Regaining” control of an uncontrolled life* that was not connected to interview 8. All the participants described their experiences of being out of control, both in everyday life and in relation to gambling. Being out of control could be experienced as very difficult, but in some situations, it could give a sense of relief. Gambling could offer both feelings of being in and out of control and entering “the bubble” in gambling could be one way of regaining control. The control experienced in and through gambling was formulated as a false sense of control, and succumbing to the reality of being out of control was an important and sometimes necessary step in seeking help. The GETs are displayed in Table II, together with their subordinate subthemes. The table also displays examples of Personal Experiential Themes (PETs) and discarded themes. The first GET is concerned with the meanings of being in and out of control and is constructed using contrasts. The second GET is also formulated through contrasts and covers the experiences of navigating different aspects of control in relation to gambling. The third GET refers to the delicate act of regaining control of the self and perceived life situation.

**Table II.** Themes and subthemes.

GETs	Subthemes
(Un)predictability and (un)safety: The meanings of being in or out of control	Security versus a false sense of control Non-control as scary versus a sense of relief
Inside “the bubble”: Navigating (no) control in and through gambling	2.1 Control as flow “inside the bubble” Experiencing a split self The “big win” and a false sense of control
A delicate act: “Regaining” control of an uncontrolled life	Artificially stitching together an uncontrolled life and self Succumbing to the reality of being out of control and seeking help.

Participants described their experiences of control in terms of predictability and safety. By contrast, being out of control was experienced as feeling uncertain and having a false sense of control. Control is generally described as something positive, but depending on the context, not having control could also be perceived as positive. In other words, being out of control could be scary and give rise to a sense of relief.

### Security versus a false sense of control

Many participants described how their experience of control involved predictability, such as planning and knowing what will happen during the day. This could include setting up routines but also overseeing and deciding what will happen on a certain day. Most people said they enjoyed having control as it gives energy:

Now I set the alarm for this time, so that I have time to take a morning walk around this lake. It takes about this long. Then I will have five minutes for meditation, and then I will have time to eat breakfast and, without stress, I will be able to get to work. And then I know I will have energy and I know I will feel good. (7)

In contrast, being out of control resulted from unpredictability and uncertainty. One participant explained:”Any situation really where things happen that I can’t control or that I haven’t planned for. That’s hard for me” (8). Feeling out of control was experienced as hard, unpleasant, and negative. An important aspect of not having control that was mentioned was losing the ability to act in a constructive way. One participant said “well, when I eat for example, then I don’t eat until I am full but until […] I can’t walk anymore!” (1). Being out of control and uncertainty could also be about being vulnerable and exposed, for example, when being informed of a serious illness of oneself or a close relative. Thus, predictability in daily routines fosters a sense of control and well-being, while unpredictability and uncertainty lead to feelings of helplessness and vulnerability.

In addition, control was experienced as feeling safe, calm, and not being stressed. Regardless of the situation, experiences of control and safety are associated with good self-esteem and competence. The experience of control can also be described as an inner balance, being able to regulate oneself in demanding situations or crises.

Although the distinction between experiencing being in control and out of control tended to be presented as clear, an intermediate position emerged in the form of a false control, which cannot be easily placed in either extreme. The experience of control was reported to be present, whereas actual control was absent. One participant said, “I want control and I imagine I have it, but really I don’t. It almost always turns out that way” (6). The realization of a more genuine absence of control could contribute to feeling more grounded, but it could also generate uncertainty, as it was realized that the experience of control is not always grounded in reality. Hence, the experience of control is closely linked to feelings of safety, self-esteem, and competence but can also involve a deceptive sense of control that may not reflect reality.

Perhaps the main contribution of this theme is that it clarifies that in order to fully examine control as a phenomenon, we need to understand the context of experiences as well as the interaction between different parts. Understanding control in this way means looking at the whole rather than just focusing on individual parts.

### Non-control as scary versus a sense of relief

In general, experiences of being out of control were described as something fundamentally difficult, negative and scary. Participants said that the experience of being out of control could give rise to worry and self-criticism.

However, while the absence of control was typically seen as negative, participants were also able to formulate exceptions where the positive aspects of being out of control were highlighted. For example, when one participant said: “Yes, I can’t influence anything. So I don’t need to do much, or I can’t do anything. I don’t need to do anything but leave it to those who can do something about the situation” (2) The experience of being out of control was associated with capitulating on a situation. Not being in control can be understood as not being responsible for the difficulty or pain of a situation. Being out of control meant handing over control to someone who then took responsibility, which in itself could be relief. This experience of handling responsibility could be linked to difficult situations in general and was not only associated with experiences specific to gambling.

Participants’ narratives revealed that control was often intertwined with predictability and a sense of safety. Knowing what will happen during the day and having the ability to plan instilled a sense of energy and well-being. This predictability, as described by many, is the cornerstone of feeling in control. Conversely, the absence of control is marked by unpredictability and uncertainty. The resulting experience was often one of distress and discomfort. This lack of control could lead to feelings of vulnerability and exposure, especially in unforeseen circumstances such as receiving serious health news. However, control experience is not always straightforward. Although generally perceived as positive, there were contexts in which the absence of control could bring a sense of relief. This duality highlights the complex nature of control, where being out of control can be both frightening and liberating. By exploring these horizons of meaning, we can gain deeper understanding of how control is experienced in relation to gambling. Through a detailed and nuanced examination of these experiences, we appreciate the multifaceted nature of control. Recognizing the interplay between predictability and uncertainty and how these factors shape the experience of control can provide valuable insights into the dynamics of gambling behaviour and serve as a meaningful framework.

### Inside “the bubble”: navigating (no) control in and through gambling

Against the background of generally experiencing being in control as a sense of predictability and safety that elicits positive feelings, gambling was described by participants as a “bubble,” they could enter to experience more control. Through gambling, they came into touch with experiences reminiscent of flow and mindfulness. However, the sense of false control and losing control inside the bubble were also experienced in relation to gambling.

#### Control as flow inside the bubble

Some participants expressed that they could turn to gambling to experience control. One participant described this experience as “when I went into my bubble and had my gambling, then I thought I had control” (5). At the same time, as this participant experienced control inside the bubble they were out of control in their everyday life. The more prominent the experiences of negative consequences and being out of control became in life outside the bubble, the stronger the incentive to step into the bubble. As such, the bubble was a safe place to go to. Inside the bubble they experienced competence and predictability, experiences described above as desirable. Participants described that the appealing aspects of feeling competent and experiencing a certain degree of predictability led them to actively seek gambling. Through gambling, they can experience an increased sense of control over their lives and disconnect the feelings related to the cumulative negative consequences that result from increased gambling. The ability to enter the bubble at any time contributed to not seeking more in-depth and long-term solutions to the problems in life, but as one participant stated:

You feel bad, because then I felt bad, I just wanted to feel good, the gambling was the medicine, and now I realize a bit more how I act myself, etc. So, when I feel bad, and I think it’s also the same for people, when I feel bad, I can, when I’ve gambled for so long, the gambling was there, if I had maybe taken drugs or something, then maybe drugs would have given me something else or I don’t know. (3)

As previously discussed, the themes of predictability and certainty have emerged as pivotal elements in the perception of control. This observation aligns with the notion that these factors are integral to the pull of the time spent within the perceived safety of the bubble that was offered by gambling. The bubble had the possibility of making the gambling environment both irrelevant and private, it was possible to enter the bubble at the toilet, in bed, at the bus, at a playland for the kids, on the lunch break, or even during work. Being in the bubble offered a state of full focus and strong presence because:

When I gamble like that, it’s just what exists right there and then. I don’t think about anything else. I don’t think about my family or consequences, or what will happen next. Instead, I’m so focused on what I’m doing on gambling. How I’m going to play the next game, what is happening in this game? So, all… What can I say? It’s almost a bit like you could compare… People sometimes say that you get into a flow if you do what you like. Whether it’s creating music or art or knitting, or whatever. And somehow, I can almost feel that when I gamble, I also get into this flow. That time stands still and there is just that. (7)

The significance of this form of complete presence is side with the previously described negative emotions that might otherwise arise from the sensation of being out of control. The state was discussed using terms such as flow and mindfulness and was associated with pleasure and peace. Thus, gambling could provide a break from external reality. Another participant described this as:

Yes, of course, it was something positive. It served a purpose in the beginning. And it was calm, I think, that I went into my bubble. That I could be in that world for a while and let my thoughts… Everything real was gone, like you can move into a little fantasy world. “If only this happens, then I can do that” and so on. Escaping reality too. Maybe there was some mindfulness in it, in a way. (9)

These positive experiences provided additional motivation to remain in the bubble and feel a sense of control.

#### Experiencing a split self

Although stepping into the bubble was described as a way to experience control, participants also described experiencing being out of control inside the bubble. Several participants were able to describe how they came to experience themselves as two different personalities in their gambling. This experience has been described as fragmented and pathological. One participant expressed: “Or almost like schizophrenic, that’s not me… The amateur psychologist, schizophrenic. That is, there are multiple personalities in the head, talking” (8). The conversations between different personalities were often described as highly conflicted about whether gambling should be continued or ended:

So, I become like. I don’t know if I should say schizophrenic. But, I become like two people. It’s like I’m talking to myself. One person says one thing, and the other says another. But it’s always the bad one that wins over the good one. But I get all. I felt completely crazy. (1)

The whole experience was disintegrated, and the experience was that there were two parts of the personality: one good part versus one bad part. The experience of oneself as two different personalities, or even two different persons, made the whole experience difficult to understand and made gambling feel unreal. It was as if the participants themselves did not understand what was happening to them, making it even more difficult to plan and limit their gambling. Participants also expressed that when experiencing being out of control during gambling itself, they were able to keep gambling to experience more control again.

Yes, but you can lose control, and then you want to regain command, so to speak, so you keep gambling, because that’s when you felt good. You thought you were going to win money. So, you could say that when you lost control, you kept going to get it back. So … You just have to get the money. (6)

This meant that participants sometimes felt that they had some control over their gambling, which gave them a sense of competence and predictability, feelings mentioned above as important to well-being. At the same time, they often experience gambling as being out of control, creating anxiety and stress. This contradiction between occasional feelings in control and simultaneous feeling out of control led them to continue gambling in an attempt to regain lost control. This created a vicious cycle in which gambling persisted, as each attempt to regain control through gambling only led to further loss of control. The combination of the possible experience of control and the present feeling of being out of control contributed in practice to a vicious spiral in which gambling could continue.

#### The big win and a false sense of control

The experience of control in gambling was valued for its own sake, but there was also an almost instrumental value as there existed a vision of the big win that would somehow sort out the chaos and solve all the problems gambling had created: “Because if I stop now, I also know that I will be in a fucking mess. But then, if I keep going, I will probably be in a shit hole, but maybe not. Because I might win” (7). Thus, it was as if the participants were not really experiencing being out of control, but were aware of the fact that they would experience becoming out of control the same moment as they stepped outside the bubble. Thus, continuing to gamble has become a means of avoiding and distancing oneself from the experience of being out of control. Here, it is possible to see how the formerly explained experience of a split desire to gamble or not gamble is seen in a new light, turned into a somewhat more profound dilemma of having control or not.

Several participants described how they previously were deluding themselves that they were in control and that they had experienced a kind of illusion of being in control over the activity itself and its consequences, for example, in terms of time spent, grip on one’s finances, or a reasonable attitude in important relationships.

### A delicate act: “regaining” control of an uncontrolled life and self

The experiences of gambling led many participants to talk about how they tried to regain control of their uncontrolled self. Some of these experiences can be interpreted as trying to stitch together a shallow facade of the controlled self. However, in the end, the participants explained that the only way of regaining control was to realize that one was out of control and needed help. The latter process was complicated by not always getting the support that the participants said they needed.

#### Artificially stitching together an uncontrolled life and self

Drawing on the participants’ experiences, one way of trying to regain control when gambling become out of control, was making sure that other areas of life were protected. For example, participants said that they worked hard to make sure that work must never be allowed to suffer and protecting family members from being negatively affected. The experience of protecting some areas of life was described as a lifeline: “Yes, work is my lifeline. Therefore, it has always been like that. It is the most important thing for me. Fortunately, I say”. (2). Simultaneously, these protected life areas were also portrayed as a type of risk element because if they were to lose that too life would no longer be worth living. Thus, this can be interpreted as a balancing act between safety and risk driven by the pursuit of control, which has been described as a highly precarious strategy for regaining control.

Many participants described similar experiences of artificially trying to regain control in relation to the shame they experienced because of gambling. Due to lies, debts, or having gambled away their own or other people’s money, the feeling of guilt and shame was enormous. Instead of facing these emotions, many described how they would continue gambling to win. One way of dealing with guilt and shame was to get even more into gambling in order to get a break from all the negativities. In the long run, however, this led to more negative experiences because:

When you step out of the bubble, it is associated with very strong feelings of anxiety, guilt, and shame. But in that moment, I block all those other thoughts. That’s what I mean by being in a bubble. There is nothing else but the gambling”. (7)

Thus, it became a vicious spiral where feelings of guilt and shame drove gambling, which in turn gave rise to even more guilt and shame. This can be interpreted as a strategy of focusing on the here and now versus the long-term. Others explained that if gambling would not resolve this vicious cycle, they would rather flee the country or commit suicide that facing reality because. One participant said:

I saw no possibility of any life afterwards. I felt so bad that it was basically a matter of life or death, I saw no solution, how am I going to solve this. Now, the Enforcement Authority was after me, I didn’t want to end up with the Enforcement Authority, I didn’t want to, and even my wife, the whole thing, we fought a lot, everyone is affected, it was, you feel so much, the shame and guilt are so great that you just want to disappear. So I think it’s very difficult, when it comes to this addiction, it has enormous consequences for people, even after they have stopped and maybe don’t gamble anymore, it still has enormous consequences. (3)

One aspect feeding into these non-constructive strategies of stitching together control was also the self-stigmatizing idea that people should get themselves together when they have lost control. Participants were able to recall their own past prejudices on gambling and how they considered problems with gambling incomprehensible before they got into it themselves.

How can it be that you win several million and then spend it? I said. Something like that. Then, I did the same thing myself. It’s hard to understand how strong that power can be if you haven’t had it yourself. That’s what’s hard to explain. (6)

Based on this, they expected that others, both people around them and the authorities, would view gambling as an indication of poor character, stupidity, and moral reprehensibility. This was a perspective they wanted to avoid simply trying to regain control by themselves. This also led many to hide their gambling and its consequences as much as possible. One participant described that “the brain is constantly on like thinking about solutions and how I’m not going to let it get out like” (7). In other words, this strategy often led to huge stress in ensuring that others would not note that they were out of control.

#### Succumbing to the reality of being out of control and seeking help

Participants described how letting go of the strategies of hiding and stitching together a facade of having control was the steppingstone to regain control of themselves and their lives.

The realization of an illusionary experience of control could come after longer periods of gambling in the context of dramatic negative consequences, such as imprisonment or contact with the bailiff. However, this realization could also be linked to treatment processes. One participant said:

Once I got into that it became addictive and more of a compulsive gambling for me, I wasn’t in control at all, even though I thought I was once I got started. But I wasn’t. I can see that afterwards, I can’t see it when I’m in the middle of it like, but it’s this unreasonable optimism that comes in like “well, I’ll fix this”. (5)

The illusion of control contributed to the subjective experience of overestimating one’s ability to cope with the negative consequences of gambling and thus became an obstacle to taking responsibility for the situation. Being transparent about gambling problems and being out of control was described as an important part of recovery:

I have taken control of my transparency and really taken control of people’s insight into my gambling. They know that I have a gambling addiction, and I am open about it […] then I feel that I have some kind of control. (4)

This process of becoming open about one’s problems was often described as difficult and experienced as a last resort when the individual had completely lost control of their life and themselves. However, it was only through this experience that the participants could come to the realization of needing help. One important step in seeking help is to put their experiences into words.

Once participants sought help, many had experienced shortcomings in the way they were treated by professionals where, for example, treatment centres were perceived not to really believe them. Several participants thought that they would have received better treatment if they had other types of addiction because there was a greater understanding of that kind of problem: “But gambling problems, you could see from their faces that it wasn’t easy to understand this “(6).

Personal experiences may be required to truly understand the complexity of gambling. Meeting others created hope, understanding and a new perspective because “going and regularly […], talking about my experience of it, and hearing other people’s experience—both of my story and of their own” has meant that “I’ve got lots of perspective” (7). Trying to understand the experiences of others became a step towards trying to understand their own experiences. Several participants felt that these perspectives provided them with tools to deal with guilt and shame. One participant said they were unsure whether they would have coped on their own as support is their medication. Regaining the sense of inner balance and being more grounded has become an important foundation in a more sustainable and long-term experience of being in control. Being in control, as mentioned earlier, is associated with the experience of being able to regulate oneself. The support of others in the same situation also allowed participants to understand “oh, I’m not alone” (10) and thus break their destructive thinking that there was something wrong with them.

## Discussion

The present study aimed to explore the lived experience of control among individuals with a history of gambling problems. Our findings indicate that being in control is generally perceived as a positive, albeit multifaceted, experience. A notable theme in the interviews was a phenomenon of false control, in which participants reported feeling in control during specific gambling episodes, despite control lacking in their wider life context. Conversely, the sense of being out of control was described as frightening, yet sometimes perceived as liberating and even beneficial. Gambling was frequently described as a “bubble” that participants could enter to regain a sense of control. Within this bubble, they experienced a state of flow, and a temporary reprieve from internal stress and/or external chaos, which is turn was caused by gambling. While gambling induced feelings of guilt and shame, entering the bubble provided a temporary relief from life’s negativity, contributing to a vicious cycle, in which gambling itself, and the “big win”, was seen as the only available solution to gambling problems. Many participants maintained protected areas of their lives—domains they kept insulated and unaffected by gambling—as lifelines that prevented total collapse. Yet, this also entailed significant risks. These compartmentalized areas of life, while offering a sense of order and stability, were highly exposed; if they were directly impacted by the negative consequences of gambling, the effects could be severe. With few remaining protective buffers, even a single disruption could lead to far-reaching and potentially devastating outcomes. Opening up and speaking honestly about the experience of being out of control was seen as a crucial step towards breaking free from this vicious cycle and achieving true control.

This study underscores the importance of situating control within the broader life context of individuals who navigate their sense of agency in the face of gambling challenges. It highlights the need for a more expansive understanding of control, complicating conventional narratives of disordered gambling as solely a collapse of agency. Instead, participants sought refuge in gambling to re-establish a sense of safety, predictability, and mindful presence, despite the destructive consequences. Control in gambling must therefore be understood not only in terms of attempts to regulate gambling behaviour, but also in relation to how individuals construct meaning in their everyday lives. Stigma, guilt, and shame contribute to the perceived need for impression management, further entrenching the complexity of control as it intersects with identity and social perception.

According to Morsi (2021), no one who starts using drugs does so with the intention of becoming addicted or losing control. This perspective is equally pertinent to gambling. Participants in this study described how, on the contrary, they turned to gambling to create an experience of control, if only for a while. They describe how they were able to step into a “bubble” where they felt competent and where life even felt understandable and predictable. This aligns with Ricketts and Macaskill’s (2003) observation that a perceived sense of achievement in gambling was often linked to the feeling of being an “expert,” reinforcing the illusion of mastery amidst escalating harm.

Participants’ experiences suggested that the more negative consequences gambling had created in everyday life and in the relationships around them, the greater the need to step into the protective bubble where only they and the gambling exist. This vicious spiral was also described by participants in Ricketts and Macaskill’s (2003) study. Clinically, these findings underscore the importance of asking not only about the loss of control associated with gambling, but also about perceived gains in control. A broadened conceptualization of control-one that includes both increased control through gambling and diminished control in everyday life—may help individuals reconcile these conflicting life domains. Future interventions could benefit from collaboratively developing alternative, sustainable “bubbles” or safe spaces that offer similar emotional regulation without the harmful consequences.

Descriptions of the experience of stepping into the “bubble” of gambling were not easily situated in neither experiences of control nor the lack thereof. In contrast, participants described how both experiences often coexisted. The experience of being in the “bubble” was perceived as pathological and alien, with an internal dialogue taking place between two voices pulling in different directions: to gamble or not to gamble. These experiences show that people with gambling problems can be aware of and realize that they are not in control and still feel compelled to gamble. While gambling can provide an immediate sense of control, over time it paradoxically leads to increased loss of control in the form of, for example, devastating debt, impaired mental health, and damage to important relationships (see Yi & Kanetkar, 2011).

The experience of being in the “bubble” closely parallels Shüll’s concept of “being in the zone,” which she describes as a state marked by intense presence, a sense of flow, and an alluring predictability—what she even terms a “dissociative bubble” (p. 196). However, the accounts from our participants reveal a more conflicted and fragmented experience within this state. Unlike Shüll’s portrayal of a woman who prepares for uninterrupted gambling by wearing dark double wool trousers to urinate without leaving the machine, our participants describe moments of inner turmoil—shouting at themselves, feeling schizophrenic, and torn between competing internal voices. These descriptions suggest a more unstable and psychologically divided experience than the immersive, almost meditative state Shüll outlines. Schüll (2012) explores the phenomenon of modern machine gambling, highlighting how “being in the zone” can lead gamblers to lose awareness of their physical bodies as they become deeply immersed in the gambling experience and its associated flow. This process of disembodiment occurs when gamblers transcend their physical selves to enter the game’s world. “being in the zone” is characterized as a trance-like state where “time, space, and social identity are suspended” (Schüll, 2012, p. 13).

Furthermore, Schüll (2012) notes that a part of the mind, while experiencing this zoned state, can remain “sharp and aware” yet lacks the capacity to act (p. 23). Although it does not correspond to the highly conflicted state described in terms of a bubble by our participants, this experience suggests that there can at least be an element of tension or conflict even within the zone. Shüll states that “as the worldly value-charge of money intrudes upon the zone, it introduces tension where tensionlessness is sought and relationality where dissociation is sought (2012, p. 200).

Being out of control was often described as being associated with negative experiences, but participants’ accounts of control in everyday life show that in some situations, it can be both reassuring and a relief to hand over control to someone else. These situations were described as instances in which participants felt that they had little control or that the control was outside themselves. Even in the deepest loss of control, there was also a seed of control present. This complexity adds a valuable contribution to research showing that gambling can be a way to create control over negative emotions in the absence of other strategies (Corless & Dickerson, 1989; Ricketts & Macaskill, 2003).

Participants’ descriptions of life inside the bubble of gambling as an experience of flow and mindfulness provide a deeper insight into how a behaviour that has major negative consequences in several areas of life can at the same time be used as a means of creating control in life. This demonstrates the complexity of the control experience. Gambling has previously been described as a phenomenon in which people overestimate their ability to influence what happens (Langer, 1975). Here, however, it seems that the participants’ understanding shows that the experience of control is more about the feeling of competence and control. It is not only about actually being able to influence an event, but also about a broader experience of being a person with insight, competence, and knowing how to behave. The possibility of entering the bubble through gambling thus became both a temporary escape from distress and a way to restore a sense of agency and competence. However, this perceived control was ultimately illusory, as it reinforced continued gambling despite mounting negative consequences—thereby contributing to a vicious spiral in which the behaviour intended to create control increasingly undermined it.

Accounts of control in gambling can also offer a partly new answer to the question, so central to addiction research, of why individuals persist in behaviours with clearly harmful consequences. Beyond the intrinsic value attributed to the experience of control, gambling was often perceived as the only possible solution to the problems it had helped create. The hope of a big win became not only a justification for continued gambling, but also the imagined escape from an increasingly difficult life situation. This dynamic illustrates a vicious spiral: gambling generates negative consequences, which in turn intensify the perceived need to gamble as a means of resolution, thereby reinforcing the cycle of harm.

An indication that a traditional understanding of control might be too narrow is the participants’ descriptions of a “false” type of control where the individual desired and partially experienced control, but where a reality-based aspect of control was absent (See also Nuske & Hing, 2013). This experience was not equivalent to the illusion of control, which refers to a false belief on the part of the gamblers that they can control the processes of gambling itself based on, for example, skill or luck. Rather, it is a question of the experience of control being present, whereas a deeper, more grounded experience of control may be marked by its absence. This was described as the case for control both in relation to gambling and everyday life.

The type of false control that the participants spoke of is different from the concept of illusion of control that often appears in literature and points to an illusion of controlling things that are, in fact, uncontrollable (cf. Langer, 1975). The false control described by the participants could lead to a failure to recognize the extent of their problems and therefore become a contributory factor in postponing seeking help for their gambling. This provides deeper insight into the widely known fact that people with gambling problems have a great reservation to seek help (Braun et al., 2014). Another reason for the postponement mentioned by the participants and in the literature is the experience of stigma surrounding gambling. A part of this process mentioned in the literature is the minimization of one’s own problems (Suurvali et al., 2009). This study shows that control can be a crucial part of that process; by overestimating one’s control over gambling, the problems are diminished, and the reservation of help-seeking behaviour remains large.

The results of this study suggest that the subjective experience of valuable relationships and arenas in life that can serve as lifelines upon their loss could turn into risk factors for suicide. For example, one participant described balancing gambling while impeccably performing at work. When holidays came and work was no longer a regulating function, the person tried to commit suicide. This shows that in treatment contacts, it is important to ask about individual risk factors, not only for relapses, but also for the risk of suicidality. Different obstacles to help-seeking behaviours are very serious, as gambling problems are associated with an increased risk of suicide. A Swedish study showed a 15-fold risk for people diagnosed with gambling problems compared to the general population (Karlsson & Håkansson, 2018). According to Blaszczynski and Farrell (1998), the highest risks may follow large losses and the disclosure of criminal activities.

Guilt and shame were recurring components of participants’ experiences (see also Yi & Kanetkar, 2011). One way to control and regulate these negative emotions is to gamble even more. The regulatory function of gambling is well studied in the literature (Corless & Dickerson, 1989), here, we can see how guilt and shame lead to more gambling, which in turn can lead to more guilt and shame, that is, how these elements seem to be part of a vicious spiral in which the regulatory function becomes active in driving gambling (see also Ricketts & Macaskill, 2003; Weatherly & Cookman, 2014). It also becomes clear that, in treatment, we may need to highlight and address self-image and stigma as possible components that perpetuate gambling.

Yi and Kanetkar (2011) found that guilt in relation to gambling problems is linked to constructive coping strategies, such as help-seeking, whereas shame is associated with avoidant behaviours aimed at short-term emotional relief. They recommend reframing shame into guilt to promote more adaptive responses—for example, shifting from “I am weak” to “I gambled excessively because I had been drinking.” In our study, however, participants did not distinguish between guilt and shame, despite their distinct psychosocial implications (Pchajek et al., 2022). Guilt typically relates to personal moral transgressions and can motivate corrective action, while shame involves internalizing others’ negative judgements and may inhibit such behaviour.

Participants described how their past prejudices about gambling contributed to self-stigma. This, together with the often-uncomprehending attitude of their environment, contributed to keeping their gambling secret as much as possible. Being open about one’s problems was seen as valuable in the recovery process, but it was also clear that it was seen as a last resort. Perceived stigma was high, both from practitioners in healthcare settings and society at large.

To fully understand control in relation to gambling, it needs to be understood in the context and relationship to life in general. However, to offer effective treatment, we also need to address negative attitudes and beliefs towards people with gambling problems that might otherwise make it difficult to reach those affected (Hing et al., 2014). Only a minority of people who experience gambling problems seek help (Braun et al., 2014), and one commonly reported barrier to seek help is shame and fear of stigma (Hing et al., 2014; Pchajek et al., 2022; Suurvali et al., 2009). According to Hing et al. (2014) stigmatization is a social process that questions the individual’s capacity to take responsibility, behavioural choices, and moral character. Stigma can be understood as a multi-dimensional construct that includes a variation of negative attitudes and beliefs about an individual (Peter et al., 2019). Stigma may be internalized as self-stigma (Corrigan & Watson, 2002) which may have an adverse effect on the subjective identity and subjects’ sense of self (Goffman, 1963).

When it comes to self-stigma, an individual’s attribution regarding the cause of a condition can lead to assumptions on responsibility. If the assumption is that responsibility is under the individual’s control, this may result in anger and punishing behaviour (Horch & Hodgins, 2008). For example, it is conceivable that an individual who would experience a great deal of control in gambling would experience greater stigma than a person who perceives themselves to have less control. It was also difficult for participants to put into words that had really affected them, which made it difficult to seek support. It was difficult to know where to start when they were unsure whether it was an addiction. The value of meeting others with personal experience was highlighted, and perhaps it is the case that you need your own personal experience to understand the complexities of being affected by widespread gambling problems. Meeting others with similar experiences not only helped to put their experiences into words, but also helped them come to the realization that they were not alone (see also Binde, 2012).

Just as someone may use alcohol to perform better socially, gambling might be a means of searching for the admiration or approval of others. Becoming a winner through gambling is an expectation that exists as long as gambling continues. It is hypothesized that addiction is about a search for validation that is not in place during childhood (Morsi, 2021). The participants in this study have experienced how they, increasingly indebted and far removed from society, have been able to pin their hopes on a big win in order to reconnect with other people and gain higher status.

The results indicate that the complex and multi-layered phenomenon of control in relation to gambling may be deepened by exploring the experiences of control in relation to everyday life and self-image. In treatment, making an individual’s different experiences of control visible may promote recovery. To have such conversations, a holistic understanding of the complexity of control is essential.

The participants talked about the experience of being criticized and judged in counselling relationships, something that has been termed “professional stigma” (Hing et al., 2016). According to Hing et al. (2016), studies on stigma and gambling are rare, and even more so studies that focus on self-stigma and individuals’ own experiences. Professional stigma in relation to gambling and how it affects help-seeking behaviour and treatment require further attention in the literature (Anderson, 2014).

The issue of control and stigma is particularly interesting, as the experience of control, both one’s own and that of others, can influence whether stigma becomes present and which response it evokes. The importance of this connection and its possible impact on the formulation of effective interventions require further investigation.

The practical implications of the study’s findings regarding a deeper and broader understanding of the experiences of control remain to be further developed. Other questions include how our understanding of these salient experiences of control can be used in outreach work, gambling companies, and budget and debt counselling. In a geographical such as the present one, implications of knowledge around the concept of control may be particularly important but also challenging, as a very large proportion of problematic gambling occurs online. Therefore, loss of control but also individuals’ attempts to regain control may be left to the individuals themselves in their private lives to a larger extent than if gambling occurs in company with others and can be monitored and regulated in physical venues. On the other hand, the interface between technical mechanisms within gambling, and the individual, can bring new possibilities of actually facilitating control through technical harm reduction tools, such as the nationwide self-exclusion service used in this setting (Håkansson & Widinghoff, 2020; Miles et al., 2023). However, in total, more remains to be understood about these implications and they go beyond the scope of the present study.

Self-exclusion tools could be further developed to address not only the behavioural aspect of gambling but also the emotional and social dimensions of control. For example, interventions might benefit from incorporating support for impression management and identity reconstruction, especially in contexts where shame and stigma are prominent. This aligns with the study’s findings, which illustrate that control is not a binary state but a complex and shifting experience. Participants described moments of false control—a perceived sense of agency during gambling episodes that masked a broader lack of control in life. This illusion of control was often sustained within the bubble, a psychological space that offered temporary relief from shame, guilt, and external chaos. In this context, self-exclusion tools might benefit from integrating support mechanisms that help individuals navigate the emotional labour of impression management and identity reconstruction. These aspects are crucial in breaking the vicious cycle where gambling becomes both the problem and the perceived solution. For example, tools that facilitate safe disclosure and reduce stigma could support individuals in moving from false to control to a more grounded experience of control—a shift that participants associated with opening up and reclaiming agency in their broader life context.

By acknowledging the protective but risky nature of insulated life domains, interventions could also help individuals build sustainable forms of control that extend beyond gambling behaviour itself. This would mean designing tools that not only block access to gambling but also support the reconstruction of identity and social belonging, which are often eroded by the shame and secrecy surrounding gambling problems

In the Swedish context, where the gambling market has recently transitioned from a state monopoly to a licence-based system, the societal implications of self-exclusion become particularly relevant. The shift has increased the availability and visibility of gambling, potentially complicating individuals’ efforts to maintain control. This raises important questions about how self-exclusion systems are designed and promoted, and whether they adequately reflect the lived experiences of those they aim to support. As recent research has begun to critically assess the limitations of self-exclusion methods, our findings contribute to this discussion by emphasizing the need for a nuanced and context-sensitive approach.

## Limitations

Individuals with gambling problems may be interested in underreporting gambling to hide behaviours that they or others might perceive as problematic (Goldstein et al., 2017). This is a complicating factor for the interviews in which this article is based and studies with a similar design. Participants described several different aspects of what could be interpreted as impression management (Goffman, 1990), including keeping their gambling secret and lying about it to manage their perception of them in their environment. Although possible under-reporting may have been the case and some things might have been withheld, the fact that the participants themselves were active and driven regarding their participation in the study may suggest that they were still willing to talk about their experiences. However, impression management can be thought of as occurring at both a conscious and a more subconscious level, so it can by no means be ruled out that different variants of underreporting also occur in the material on which this article is based.

Recruiting participants only through peer support groups means that only those who have some kind of connection to these groups can participate. For example, this may exclude people who opt out of peer support contexts. There may be several reasons for not participating in such groups, with research showing, among other things, that participation insinuates that you cannot solve your own problems, that there is no group around and/or that you are not very social (Hing et al., 2016). This limits the transferability of the results. Another factor that may affect transferability is that most of the participants cohabited with a partner, which has previously been shown not to be characteristic of people who experience gambling problems.

## Supplementary Material

Clean copy ZQHWS20250015.docx

## Data Availability

The data that support the findings of this study are qualitative and contain sensitive information that could potentially indirectly identify the participants if read in totality. Therefore, to protect the privacy and confidentiality of the participants, the whole data set is not publicly available. Further details about excerpts supporting the results of analysis can be obtained from the corresponding author upon reasonable request.
